# Supplementary material for: Preference of cesarean delivery and its associated factors among pregnant women attending ante natal care at public health facilities of Debrebrehan City, Ethiopia: Cross-sectional study
Source: PLoS One. 2024 Jan 31;19(1):e0296990. doi: 10.1371/journal.pone.0296990 (PMC10829985; doi:10.1371/journal.pone.0296990)
Supplement: S1 File — (DOCX) [file pone.0296990.s001.docx]

Questionnaire (English version)

General information

1.Date of data collection ______________

2. Study ID code _________________

4. Place of Data collection______________________

Hospital___________________

Health center________________________

| S.N | Questions | Options |
| --- | --- | --- |
| 101 | Where is Your residence? | 1. Urban 2. Rular |
| 102 | How old Are you? | ________Years |
| 103 | What is your current marital status? | 1. Single 2. Married 3. Divorced 4. Widowed |
| 104 | What is your educational status (level)? | 1. No formal education 2. Primary education 3. Secondary education 4. College and above |
| 105 | What is your partner’s educational status (level)? | 1. No formal education 2. Primary education 3. Secondary education 4. College and above |
| 106 | What is your occupation? | 1. Government employee 2. NGO employee 3. Private employee 4. Merchant 5. Student 6. House wife 7. Farmer 8. Other ------------- |
| 107 | What is your partner occupation? | 1. Government employee 2. NGO employee 3. Private employee 4. Merchant 5. Student 6. Farmer 7. Other --------- |
| 108 | Average family Monthly income | ------------------birr |

| **2. Obstetric factors** | | | |
| --- | --- | --- | --- |
| S/N | Question | Option | Skip |
| 201 | How many times have you ever been pregnant? | ____times |  |
| 202 | Have you had spontaneous abortion previously? | 1. Yes 2. No |  |
| 203 | How many times have you ever been deliver? | ________times |  |
| 204 | How many live children do you have? (in number) | ______________ |  |
| 205 | Have you previously had any health problems related to pregnancy? | 1. Yes 2. No | If the answer is no please skip 206 |
| 206 | If yes, please specify | --------------- |  |
| 207 | Have you had one CS previously? | 1. Yes 2. No |  |
| 218 | What was the previous mode of delivery? | 1. SVD 2. C/S |  |
| 209 | What was previous place of delivery? | 1. Home 2. health center 3. Hospital 4. Private institution |  |
| 210 | Is the current pregnancy planed? | 1. Yes 2. No |  |
| 211 | Do you have encounter any obstetric complication in the current pregnancy? | 1. Yes 2. No | If the answer is no please skip 216 |
| 212 | If Yes for the above question ,Please specify the problem. | ------------------------------------------------ |  |
| 213 | Gestational age ( in weeks) | -------------- |  |
| 214 | How many ANC visit do you have in the current pregnancy? | _______________ANC visit |  |
| 215 | Did you discussed with your partner about mode of deliveries? | 1. Yes 2. No |  |
| 216 | Did your partner support you with your decision regarding 220mode of delivery? | 1. Yes 2. No |  |
| 217 | Where do you prefer to deliver for the current pregnancy? | 1. Public Hospital 2. Public Health Center 3. Private Clinic |  |
| 218 | Have you ever counselled on mode of delivery from health care provider? | 1. Yes 2. No |  |
| 219 | Do you have a close friend or family member who has delivered by cs? | 1. Yes 2. No |  |
| *220* | Do you have full freedom to decide about your mode of delivery? | 1. Yes 2. No |  |

| 3.Maternal satisfaction in previous mode of delivery | | | | | | |
| --- | --- | --- | --- | --- | --- | --- |
|  | strongly dissatisfied =1 , dissatisfied=2, Neutral=3 satisfied=4 strongly satisfied5 | | | | | |
| S/N | Questions | 1 | 2 | 3 | 4 | 5 |
| 301 | Availability, accessibility, and cleanliness of toilet |  |  |  |  |  |
| 302 | Cost of services |  |  |  |  |  |
| 303 | Respectful (by birth attendants) |  |  |  |  |  |
| 304 | Verbally encouragement by birth attendants |  |  |  |  |  |
| 305 | Adequacy of time spent with you by birth attendants |  |  |  |  |  |
| 306 | Delivery position |  |  |  |  |  |
| 307 | Privacy of delivery care processes |  |  |  |  |  |
| 308 | Welcoming by birth attendants |  |  |  |  |  |
| 309 | Pain management |  |  |  |  |  |
| 310 | Allowing families on your side |  |  |  |  |  |

| **Part 4 Knowledge assessing questions** | | |
| --- | --- | --- |
| S/N | Questions | Option |
| 401 | Cesarean delivery is less painful? | 1. Yes 2. No |
| 402 | Maternal complications of cesarean delivery are greater | 1. Yes 2. No |
| 403 | Infection risk of cesarean delivery is higher than vaginal delivery | 1. Yes 2. No |
| 404 | Emotional relationship between mother and baby after vaginal delivery is better | 1. Yes 2. No |
| 405 | Infants born by CS are good compared with those born by vaginal delivery | 1. Yes 2. No |
| 406 | Infant bone fractures are impossible in CS | 1. Yes 2. No |
| 407 | Caesarean section delivery is less complication for babies as compared to vaginal delivery | 1. Yes 2. No |
| 408 | Respiratory disorders in infants born by CS are less than vaginal delivery | 1. Yes 2. No |
| 409 | Hemorrhage after cesarean delivery is less than vaginal delivery | 1. Yes 2. No |
| 410 | CS is reasonable when the baby is in breech presentation | 1. Yes 2. No |

| **Part 5: Attitude scores of antenatal mothers attitude towards caesarean delivery**  strongly disagree =1 , disagree=2, Neutral=3 agree=4 strongly agree 5 | | | | | | |
| --- | --- | --- | --- | --- | --- | --- |
| SN | Questions | 1 | 2 | 3 | 4 | 5 |
| 501 | Caesarean section is better than vaginal delivery. |  |  |  |  |  |
| 502 | Would prefer caesarean section because I don’t like to go through all the position and straining of vaginal delivery. |  |  |  |  |  |
| 503 | Would prefer caesarean section because I don’t like to go through labour pain. |  |  |  |  |  |
| 504 | Baby born by caesarean are more healthy |  |  |  |  |  |
| 505 | CS is better because we can undergo tubal ligation at same setting. |  |  |  |  |  |
| 506 | CS is better because prevents bladder and Uterine prolapse. |  |  |  |  |  |
| 507 | CS is better because it prevents deformation and tear in genital tract. |  |  |  |  |  |
| 508 | I would prefer caesarean section even with its inherent complications. |  |  |  |  |  |
| 509 | CS should be performed as a choice of the mother) |  |  |  |  |  |
| 510 | CS should be performed when vaginal delivery is risky |  |  |  |  |  |

| **6. Preference of mode of delivery** | | | |
| --- | --- | --- | --- |
| **S/N** | **Questions** | **Options** | **Skip pattern** |
| 601 | Have you ever been planning about your mode of delivery? | 1.yes  2.no |  |
| 602 | If you are given the freedom to decide alone, which mode of delivery do you prefer? | 1.vaginal  2.Caesarean section | If the answer is 1 skip Q 603 or If 2 please skip question 604. |
| 603 | Why you preferred C/S? *(More than one answer possible)* | 1.less Labour pain  2.Avoidance of emergency cesarean section  3.Safer for women  4. Less risk of fetal distress  5. A chance to choose specific date  6.quick restoration for sexual activity  7. A fashion  8. Prior negative experience from vaginal delivery  9. Health care providers were not encouraging and reassuring during previous vaginal delivery  10. Fear or the need to avoid episiotomy,  11. other |  |
| 604 | Why you preferred SVD ? *(More than one answer possible)* | 1. Natural process 2. Faster recovery 3. Healthier babies 4. Less pain after delivery 5. Easier breast feeding 6. No scar 7. Shorter hospital stay 8. No operative or anaesthetic risk 9. Lower risk of morbidity and mortality 10. No parity limits 11. Less costly 12. Health care providers encouragingduring labour 13. Others |  |
