# Supplementary material for: Preference of cesarean delivery and its associated factors among pregnant women attending ante natal care at public health facilities of Debrebrehan City, Ethiopia: Cross-sectional study
Source: PLoS One. 2024 Jan 31;19(1):e0296990. doi: 10.1371/journal.pone.0296990 (PMC10829985; doi:10.1371/journal.pone.0296990)
Supplement: Attachment — Submitted filename: Manuscript Reviewed.pdf [file pone.0296990.s005.pdf]

# PREFERENCE OF CESAEREAN DELIVERY AND ITS ASSOCIATED FACTORS AMONG PREGNANT WOMEN ATTENDING ANTE NATAL CARE AT PUBLIC HEALTH FACILITIES OF DEBREBRHAN CITY, ETHIOPIA, 2023.

**Lemlem Zewudu[1], Fetene Keshaun, Mulualem Silesh & Mitiku Tefera4*[2], Eyob Ketema Bogale[3] & Aberham Demis [4] Zewedie Yeshaw Tekle[5]**

Author's address

1. Debre Berhan compressive specialized Hospital Amhara region, Debre Berhan, Ethiopia.

2. Department of Midwifery School of Nursing and Midwifery, Asrat woldeyes health science campus, Debre Berhan University, Ethiopia.

3. School of Public Health, College of Medicine and Health Science, Bahir Dar University, Bahir Dar, Ethiopia.

4. Abrham Demis- Department of Midwifery, Deber-Berehan Health Science College, Debre Berhan, Amhara, Ethiopia.

5. Zewedie Yeshaw Tekle- Department of Midwifery, Deber-Berehan Health center, Debre Berhan, Amhara, Ethiopia.

# ABSTRACT

**Introduction**: Caesarean section is a surgical procedure used to prevent or treat life-threatening maternal or fetal complications. Women's delivery preferences have become a global issue of interest to many researchers and clinicians, especially given the ever-increasing rate of caesarean sections. There is limited data on the preference of caesarean delivery for Ethiopian women particularly in the study area. The aim of the study to assess preference of caesarean delivery and its associated factors among pregnant women attending antenatal care at public health facilities of Debre Berhan city, Ethiopia 2023.

**Commented [CP1]:** It would be okay you bring out factors within this line

**Methods**: An institution based cross-sectional study design was done from May 5-20, 2023 among 512 participants and a multi stage sampling technique was used. The data was collected by using an interviewer administered semi structured questionnaires. The data was entered by Epi Data version 4.6, then transferred to SPSS version 25 for analysis. With logistic regression those variables with a p-value <0.25 in the bi variable analysis was candidate to multivariate logistic regression and variables with a p-value <0.05 was considered statistically significant.

**Result:** The preference of cesarean section was, 133 (26%) with CI (22.3%, 29.9%). Women with previous satisfaction on intra partum care (AOR; 6.3 CI=(3.5-11), not knowledgeable to caesarean delivery(AOR;2.9; 95%CI=1.6-5.3),history previous spontaneous abortion(AOR;3.1; 95%CI=(1.5-6.3) residence(AOR;1.9 95%CI=(1.0-3.5) and current pregnancy related problem(AOR;4.9 95% CI=1.9-10) were significantly associated with Preference of caesarean delivery.

**Commented [CP2]:** It would be good to indicate P-value as a measure mentioned for significance

**Conclusion and recommendation:** In this study the preference of cesarean delivery was high as compared to world health organization recommendation. Previous Satisfaction on intra partum care, current pregnancy related obstetric problem, Knowledge of the Respondents towards caesarean delivery, Previous spontaneous abortion, Residence were significantly associated with Preference of caesarean delivery. Designing strategies to enhance maternal satisfaction by strengthening adherence to intra partum care.

**Commented [CP3]:** Satisfaction on what?


**Key words:** Preference of cesarean delivery, cesarean delivery.

# Introduction

Maternity delivery is one of the most important health care services in all countries [1]. Preference of caesarean delivery is defined as choosing caesarean section as a mode of delivery [2].Women's preferences for mode of delivery have emerged as a global subject of interest to many researchers and clinicians especially with the steady increase in the rate of cesarean section (CS), Even though the World Health Organization (WHO) advises a maximum of 10-15% acceptable rate of caesarean sections [3]. With some data showing caesarean section rates above 15% are not linked to further declines in maternal and neonatal mortality and morbidity [4].

The right to prefer mode of delivery is a crucial component of compassionate and respectful care in modern obstetrics as it fosters both maternal and neonatal well-being[5]. Caesarean section is a surgical procedure used to prevent or treat life-threatening maternal or fetal complications [6]. Pregnant women are normally involved in decision-making process concerning mode of delivery and many factors affect their decision. These processes are influenced by person's environment, values, personality, knowledge and insight which influence each other interactively[7]

**Commented [CP4]:** This statement would be good in the opening paragraph

Caesarean delivery at the mother's request (CDMR) is a branch of elective cesarean sections performed not according to medical indication, but at the mother's request [8].

C-section has become a prominent indicator of accessing progress in emergency obstetric care, and a method to avert complications during labor and delivery[9]. Modern obstetric practice has seen increases in primary CS rates everywhere for medical, social, economic, and legal reasons [10]. For instance, studies done Port Elizabeth has the highest CS rates (55.6%)[11], Latin America (40.5%), [12] in southern India region (32 %)[13], in south Africa the CS rate is (42.4%),[14], and in Ethiopia also the CS rate between 20.2 and (38.3%) of mothers were undergone caesarean section [15-17].

In Ethiopia, between 2000 and 2016, there was a slight increase in the national cesarean section rate from 0.7% in 2000 to 1.9% in 2016 [18]. Based on various attributes, differences continued to exist. Compared to rural areas, which had a caesarean section rate of 0.9%, urban areas had a

caesarean section rate of 10.6% in 2016 [18-20]. Determining their preferences in mode of delivery will help reduce the maternal and prenatal morbidity and mortality (19).

Advancement of delivery care including caesarean delivery has greatly improved the outcomes of birth globally with significant reduction of maternal morbidity and mortality. However, the evidence to support this is limited in Africa especially Ethiopia and more particularly, in the study area. The study aims at examining the factors influencing women's preferred mode of delivery.

**Commented [CP5]:** Aimed to

**Commented [CP6]:** This would be good for a qualitative study; consider other words

**Commented [CP7]:** Preference of which mode of delivery

# Methods and Materials

## Study area

This study was conducted in selected public facilities in Debre Berhan town of North Shoa, Amhara, Ethiopia. Debre Berhan is located 130 km far from Addis Ababa, and 695 Km far from Bihar Dar capital city of Amhara Regional state. The total population of Debre Berhan town is 202,226 from the total population 106,388 are females and 6,815 are pregnant mothers. According to zonal health Department report, Debre Berhan, town, has ten public health facilities which are 2 government hospitals (of which are Debre Berhan comprehensive specialized hospital and Hakim

Gizaw hospital) and 8 are health centers, which are Debre Berhan health center, Tebase health center, Ayer tena health center, Chacha health center, keyt health centers, Goshebado health center Debre, and Enkulal koso health center.

## Study design and period

Institution based cross-sectional study design was conducted,  in Debre Berhan town public health facilities, from May 5-20, Ethiopia 2023.

**Commented [CP8]:** Employing qualitative or quantitative method

## Source of population

The source population all pregnant mothers who attended their ANC at public health institution in Debre Berhan city.

## Study population

The study population all pregnant mothers who attended their ANC at selected health institutions in Debre Berhan city.

## Inclusion Criteria

All pregnant women who had one or more than one delivery and who attend the ANC during the data collection period.

### Exclusion criteria:

Pregnant mothers, who had previous CS scars, uterine rupture.

**Commented [CP9]:** Why? Is it relevant

### Sample size determination

**For the first objective (outcome),** a single population proportion formula is used to calculate the sample size by considering the following statistical assumptions: P = proportion of Preference rate of CS among Pregnant mothers from other study, 28.9% Harar Regional State, Eastern Ethiopia[21]. ( Z α/2 = Z score of 95% CI, d= Margin of error (5%). $n = \frac{(Z_{\frac{\alpha}{2}})^2 \times p(1-p)}{(d)^2}$.

**Commented [CP10]:** Did you have more than one population

n = (1.96)2 *0.289*0.711/ (0.05)2 = 316* 1.5=474 Then after adding 10 % non-response rate, the sample size was 522.

## Sampling technique and procedure

Multi-stage sampling technique was used to select representative sample. There are 10 public health facilities in Debre Berhan city; from these, 5 were selected using a simple random sampling method. The selected health facilities are Debere-Berhan Comprehensive Specialized Hospital (DBCSH), Debre Berhan health center, Tebase health center, Chacha health center and Keyt health center. The sample was allocated proportionally for each Health facilities and allocation done by using average monthly ANC follow up and which was 2100. Study participants was selected using a systematic random sampling technique. First, determine the sampling interval

**Commented [CP11]:** Did all facility leaders consent for their facility names to be published

(K) value by dividing the total pregnant women attending antenatal care at in the study period by the total sample size, which gives 2.01 ≈ 2.

Probability allocation sampling technique was to select (nf x n)/N= (Sample final * no of total pregnant women attending antenatal care in each Health facilities/ number of total pregnant women attending antenatal care within two weeks (Fig 1).

Where N is equal to 1050.

- Debre Berhan comprehensive specialized hospital = 522x 400/ 1050 = 198
- Debre Berhan Health Center = 522 x 220/ 1050 = 110
- Tebase Health Center = 522 x 150/1050 = 74
- Cacha Health Center = 522 x 176/ 1050= 88
- Keyt Health Center =522 x 164/1050= 81

## Data collection methods

Data was collected using semi structured questionnaires adapted from review of relevant literatures [22-26]. All questions were written in English language and translated in to Amharic the (local language) and then back to English by two different language experts to check for consistency and clarity. The questionnaire is divided into six sections (1-5) to obtain data on the socio-demographic characteristics of the respondents, obstetric factors, Knowledge towards Cesarean delivery, Attitude towards cesarean delivery, and Preference of caesarean delivery parts are contained.

## Variables of the study

### Dependent variable

Preference of Cesarean delivery.

### Independent variable

**Socio demographic factors**

**Obstetric factors**

**Knowledge towards caesarean delivery**

**Attitude towards caesarean delivery**

**Maternal satisfaction on previous intra partum care**

## Operational definitions

**Preference of cesarean delivery**: implies patient choice of caesarean delivery without any fetal and maternal indication [27].

**Maternal Knowledge**:- Maternal knowledge towards cesarean delivery, the questions are adapted.1 point was given to each correct response and 0 points to incorrect and 'I don't know' answers. The overall maternal knowledge score was described as good (7–10), intermediate (4–6), and poor (0–3) [26].

**Attitude towards cesarean delivery**

The questionnaire for attitude assessment was served in Likert scale format with strongly agree (score 5), agree (score 4), neutral (score 3), disagree (score 2) and strongly disagree (score 1).

Attitude to CD was assessed with 10 statements for caesarean delivery. A median attitude score was computed for each respondent for all the statements to find the overall attitude of women towards that mode of delivery. A median attitude score of 3 or less was considered as a negative attitude and a score of more than 3 was considered a positive attitude towards that particular mode of delivery [28].

**Maternal satisfaction** is the satisfaction of mothers during service delivery. The level of satisfaction was assessed on a 5-point Likert scale (1, very dissatisfied; 2, dissatisfied; 3, neutral;

4, satisfied; 5, very satisfied).1 point was given to satisfied and 0 points to unsatisfied. Those who were satisfied with ≥75% of the items were categorized as ‘satisfied’ (those who responded very satisfied, satisfied or neutral) and those who were satisfied with <75% of the items were categorized as ‘unsatisfied’ (those who responded dissatisfied or very dissatisfied [29].

## Data Quality Assurance

Five diploma midwives were participated as data collectors and one BSC midwife was controls the overall activity of a data collection method as a supervisor. One days of training was given for data collectors on objective of the study. Pre-tested on similar set of respondents was done in Debre Sina primary hospital. It was done, to check for the reliability, validity, appropriateness of format, wording and time needed to fill the questionnaire.

**Commented [CP12]:** Who participated to validate the tool?

**Commented [CP13]:** Is this necessary

## Data processing and analysis

Following completion of the data collection, questionnaires was checked for completeness and consistency, and data was entered using Epi Data version 4.6, then transferred to SPSS version 25 for analysis. Binary and multiple logistic regression analyses was performed. Variables with a p-value of 0.25 in the bi variable analysis was considered for the multivariate analysis to control the effect of confounding variables. Variables with a p-value greater than 0.05 was fitted to the multi-variable model. The odds ratio along with a 95% confidence interval (CI) was computed to ascertain the strength of association between the explanatory and outcome variables.

**Commented [CP14]:** This was to do what?

The regression model fitness was checked by the Hosmer Lemshow goodness test =0.077 and Nagelkerke R square = 0.463, and. Multi collinearity assumption was checked by Variance Inflation Factor (VIF) and there is no multi collinearity.

## Ethical considerations

Informed written consent was obtained from each study subjects after clear explanation about the purpose of the study. An official letter of cooperation was obtained for each selected Health facilities from Debere-Berhan University Asrat Woldeyes Health Science campus (protocol

number IRB-135).We considered and agreed on the beneficence, no maleficence, and autonomy of the participants before beginning data collection by obtaining consent from all chief executive officers of the hospital and health center head. The purpose of the study was explained to the study participants; confidentiality was ensured. At all levels, officials were contacted and permission has been secured.

**Commented [CP15]:** Were

# Results

## Socio-Demographic Characteristics of Respondents

The participant's level of response was 98% (512). The age of the mothers ranges from 18-45 years old with a mean age of 32.9 years. The marital status of the participants revealed that 431(84.4%) of them were married during the period of data collection. Moreover, 30.5% of respondents reported to have completed primary education,(Table 1).

**Commented [CP16]:** Good, just make it more summarized & use % only in description. The rest are in the table making 2 lines or 2 &1/2

**Table1.Socio demographic characteristics of pregnant women attending ANC in selected Public health facilities in Debre Berhan, Ethiopia, 2023.**

| Variables | Category | Frequency | Percentage (%) |
|---|---|---|---|
| Residence | Urban | 337 | 65.8 |
| | Rural | 175 | 34.2 |
| Age | 18-25 | 113 | 22 |
| | 26-35 | 283 | 55.3 |
| | 36-45 | 116 | 22.7 |
| | Single | 37 | 7.2% |

| | | | |
|---|---|---|---|
| Marital status | Married | 431 | 84.2% |
| | Divorced | 31 | 6.1% |
| | Widowed | 13 | 2.5% |
| Income | >2500 | 145 | 28.3% |
| | 2500-4000 | 120 | 23.4% |
| | 4001-10000 | 135 | 26.4% |
| | >10000 | 112 | 21.9% |
| Occupation | Employed | 166 | 32.4% |
| | Un employed | 346 | 67.6% |
| Educational status | No formal education | 143 | 31.3% |
| | Primary education | 131 | 28.7% |
| | Secondary education | 67 | 14.7% |
| | College and above | 11 | 25.4% |
| Partners Educational status | No formal education | 143 | 22.7% |
| | Primary education | 131 | 13.1% |
| | Secondary education | 67 | 25.6% |
| | College and above | 116 | 13.1% |
| Occupational status | Employed | 174 | 34% |

| | | | |
|---|---|---|---|
| | Un employed | 338 | 66% |

## Obstetric characteristics of the respondents

Among 512 respondents 94(18.4%) and 418(81.6%) were primi para and multi para respectively. The age of the respondents ranged from18 to 45 years old. From that 63 % were delivered at Health center in the previous childbirth. The majority of the respondents 352(70%) had planned pregnancy 15.8% of participants previously had pregnancy-related complications and 6.1% were currently had pregnancy-related complications. On the other hand, 46.8% of respondents reported to have a close friend or a family member who has delivered through cesarean section, and pregnant women's freedom of deciding about the mode of delivery have shown that 72.5% of respondents disclosed that they have a freedom to decide their mode of delivery. From the respondents (82.8%) have ever been planning about their mode of delivery. From the participants 129(25.2%) pregnant mothers were not satisfied with previous intra partum care (Table 2).

**Commented [CP17]:** Good!, however,
Be precise and avoid being too wordy in the description

**Table 2: Obstetric characteristics of pregnant women attending ANC in selected Public health facilities in Debre Berhan, Ethiopia, 2023**

| Variables | Category | Frequency | Percentage (%) |
|---|---|---|---|
| **Previous history of spontaneous abortion** | Yes | 78 | 15.2 |
| | No | 434 | 48.8 |
| **Current number of ANC contact** | FIRST | 234 | 45.7 |
| | SECOND | 101 | 19.7 |
| | THIRD | 49 | 9.6 |
| | 4th VISIT | 46 | 9.0 |
| | > 4th visit | 82 | 16.0 |

| | | | |
|---|---|---|---|
| **Parity** | Primi para | 94 | 18.4 |
| | Multi | 418 | 81.6 |
| **Previous pregnancy related problem** | Yes | 66 | 12.3 |
| | No | 449 | 87.7 |
| **Planed pregnancy** | Yes | 352 | 68.8 |
| | No | 160 | 31.3 |
| **Current pregnancy related problem** | Yes | 36 | 7 |
| | NO | 476 | 93 |
| **Discussion with Partners** | Yes | 306 | 59.8 |
| | No | 206 | 40.2 |
| **Partner's support to preference** | Yes | 294 | 57.4 |
| | No | 218 | 42.6 |
| **Previous intra partum care satisfaction** | Yes | 383 | 74.8 |
| | No | 129 | 25.2 |
| **Attitude towards CD** | Positive attitude | 369 | 70.9 |
| | Negative attitude | 149 | 29.1 |
| **Knowledge towards CD** | Not knowledgeable | 199 | 38.9 |
| | Intermediate | 109 | 21.3 |
| | knowledgeable | 204 | 39.8 |

### 6.3. Preference of caesarean Delivery

To determine women's preference for Cesarean delivery, 133 (26%) with CI (22.3%, 29.9%) of the respondents prefers caesarean delivery as a mode of delivery (Figure 2).

### 6.4. Reasons for preference of cesarean delivery

From the mothers preferred that cesarean delivery 99(24.8%) because of CS has less Labour pain (Table 4).

**Table 4: Reasons behind Women's Preference' for Caesareans delivery Among pregnant women attending ANC in selected Public health facilities in Debre Berhan, Ethiopia, 2023**

| Variable | Frequency | Percentage |
|---|---|---|
| CS has less Labour pain | 99 | 24.8 |
| Avoidance of emergency cesarean section | 22 | 5.5 |
| Safer for women | 69 | 17.3 |
| Less risk of fetal distress | 41 | 10.3 |
| A chance to choose specific date | 9 | 2.3 |
| quick restoration for sexual activity | 12 | 3.0 |
| A fashion | 42 | 10.5 |
| Prior negative experience from vaginal delivery | 55 | 13.7 |
| Health care providers were not encouraging and reassuring during previous vaginal delivery | 10 | 2.5 |

**Commented [CP18]:** You could insert a figure like pie chart, etc as appropriate

**Commented [CP19]:** Could be extracted from the table

| | | |
|---|---|---|
| Fear or the need to avoid episiotomy | 31 | 7.8 |
| Other | 9 | 2.3 |

**6.4. Factors associated with Preference of caesareans Delivery**

In bivariate analysis showed that Knowledge of the respondents towards CS, Previous Satisfaction during intra partum care , Residence, Marital status , Occupation ,Planed pregnancy ,Previous spontaneous abortion, current pregnancy related obstetric problem and Discussion with partners about mode of delivery were factors associated with preference of caesarean delivery (p-value less than 0.25) and added to multivariable logistic regression analysis. In multivariate logistic regressions, Previous Satisfaction on intra partum care, current pregnancy related obstetric problem, Knowledge of the Respondents towards caesarean delivery, Previous spontaneous abortion, Residence were significantly associated with Preference of caesarean (p-value less than 0.05).

**Commented [CP20]:** Good summary

The result showed that pregnant women who lived in urban residence were 1.9 times more likely to preferred CS as compared with women who lived in rural. (AOR=1.9(1.03-3.5) P=0.038*).

Pregnant women who had previous abortion were 3 times more likely prefers CS compared to pregnant women who had no previous spontaneous abortion(AOR=3.1(1.5-6.3) P=0.001*).

Pregnant women dissatisfied in previous intra-partum care preferred CS for the current pregnancy as a mode of delivery, the degree of preference increased 6 times as compared to women who was satisfied. (AOR=6.3(3.58-11.29) P=0.01*

The other variable that was found to have significant association were knowledge of respondents about caesarean delivery, pregnant women who had no knowledge About caesarean delivery are 2.9 times more likely prefers CS as compared to had knowledge about caesarean delivery (AOR= 2.9(1.6-5.3)P=0.01*).

Pregnant mothers who had current pregnancy related obstetric problem are 4.8 times more likely prefers CS as compared to mothers who haven't.(AOR=4.8, CI=(1.9-10),P=0.001)(Table 5).

**Table 5: Factors associated with Preference of caesarean delivery Caesareans Delivery Among pregnant women attending ANC in selected public health facilities in Debre Berhan, Ethiopia, 2023.**

| Variables | Category | Preference of cesarean deliver | | COR (95%CI) | AOR(95% CI) | P VALUE |
|---|---|---|---|---|---|---|
| | | Yes | No | | | |
| **Knowledge of the respondents** | Not Knowledgeable | 66 | 133 | 2.2(1.4-3.5) | 2.9(1.6-5.3) | 0.001* |
| | Intermediate | 30 | 79 | 1.714(0.9-2.9) | 2.4(0.22-5.0) | 0.312 |
| | Knowledgeable | 37 | 167 | 1 | 1 | |
| **Previous intra partum Satisfaction** | Satisfied | 73 | 56 | 7 (4.5-10) | 6.3(3.5-11) | 0.001* |
| | Not Satisfied | 60 | 323 | 1 | 1 | |
| **Residence** | Urban | 105 | 232 | 2.3(1.4-3.7) | 1.9(1.0-3.5) | 0.038* |
| | Rural | 28 | 147 | 1 | 1 | |
| **Occupation** | Employed | 64 | 102 | 2.5(1.6-3.7) | 0.5(0.26-1.07) | 0.77 |
| | Non Employed | 69 | 277 | 1 | 1 | |
| **Planed pregnancy** | Yes | 110 | 242 | 2.7(1.6-4.4) | 1.07(0.4-2.3) | 0.858 |

| | | | | | | |
|---|---|---|---|---|---|---|
| | No | 23 | 137 | 1 | 1 | |
| **Previous history of spontaneous abortion** | Yes | 48 | 30 | 6.56(3.9-10.9) | 3.1(1.5-6.36) | 0.001* |
| | No | 85 | 349 | 1 | 1 | |
| **Maternal Education** | No formal Education | 19 | 135 | 0.1(0.6-0.21) | 0.10(0.03-1.02) | 0.112 |
| | Primary Education | 21 | 135 | 0.129(0.69-0.229) | 0.1(0.46-1.2) | 0.431 |
| | Secondary Education | 36 | 63 | 0.4(0.2-0.8) | 0.4(0.2-1.3) | 0.325 |
| | College and above | 57 | 46 | 1 | 1 | |
| **Current Pregnancy related problem** | Yes | 25 | 11 | 7.7(3.6-16) | 4.8(1.9-10) | 0.001* |
| | No | 108 | 368 | 1 | 1 | |
| **Discussion with partner** | Yes | 88 | 218 | 1.4(1.9-2.1) | 0.8(0.4-1.4) | 0.545 |
| | NO | 45 | 161 | 1 | 1 | |

# Discussion

Women's delivery preference is a subject that is widely researched and debated in many parts of the world. Women's autonomy, their satisfaction with childbirth and their active participation in the decision-making process regarding the way they want to give birth to their children are becoming increasingly important. In Ethiopia very little is known about women's preference for

delivery methods and there is no evidence on mothers' preference for caesarean section. Although CS rates in Ethiopia have also increased[30].

**Commented [CP21]:** It would be good you state what you had set out to do.

**Commented [CP22]:** It would be good you state what you had set out to do.

The purpose of this study was to assess maternal preference, cesarean delivery, and associated factors in public health facilities in Debre Berhan, Ethiopia. From this study, the prevalence of cesarean delivery among pregnant women attending ANC in public health facilities was 26%. Similar studies have been conducted in Ethiopia and other countries. This result is almost similar to the study in the southern part of Ethiopia and in Harer, which is 24.6% and 28%, respectively [21, 31].

**Commented [CP23]:** It would better fit in the first paragraph

The similarity may be that both studies were conducted during the ANC visit. However, this study result showed a higher preference for CD compared to other studies conducted at the University Hospital of Asyut, Egypt, in six European countries (Belgium, Iceland, Denmark, Estonia, Norway and Sweden) where the preference for C/S was 12.2% [22, 32]. The discrepancy emerged when this study interviewed mothers in the ANC unit while the reference studies were conducted in delivery units, which may reduce the tendency of mothers to choose CD because of fear of childbirth.

**Commented [CP24]:** You could summarize it in the previous paragraph

In this study maternal satisfaction in delivery care services were significantly associated with preference of cesarean delivery. Current pregnant mothers who delivered previously through SVD and dissatisfied with previous intra partum care were 6 times more likely preferred CD at current pregnancy. This was in line with studies conducted in Debre Markos, Gamo Gofa Zone, and southwest Ethiopia [33-35].This could be because of those who delivered through SVD may be experience with labor pains. However, for those who have delivered by CS, may be the anesthesia relieves the pain of labor and the surgery results with satisfied.

**Commented [CP25]:** Be precise and straight forward

Further, according to the findings mothers who had previous spontaneous abortion as found to have a statistically significant relationship with preference of CD. Mothers who had previous spontaneous abortion now preferred 3 times more likely CD than mothers who hadn’t spontaneous abortion. This result was slightly similar with another study that was conducted in Iran( OR =1.7) [36].

There is also association between preference of cesarean delivery and residence. The findings showed that Also, living in the urban settlement was significantly associated with the preference for CD. Respondents living in the urban areas had higher odds for the preference of CD compared to rural dwellers (odd ratio of 1.9). This is in line with other studies conducted in Ghana, Nepal, and Bangalore [37-39]. These could be urban women's are more likely to be more educated and hears about the CD, also are financially able to afford the increased costs of a CD. In addition, living in an urban settlement also improves access to quality medical facilities that are well-equipped to safely perform CS.

The other variable that was significantly associated to maternal preference of CD was current pregnancy related obstetric problem. Pregnant mothers who current pregnancy had related obstetric problem are four times more likely prefers CS as compared to mothers who hadn't. This finding is almost similar to a study conducted in Hawassa [24].This might be due to fear of intra partum complication.

**Commented [CP26]:** Not needed as it is on the result. Just start with **Current**

Pregnant mothers who had no knowledge to wards cesarean delivery were two times more likely prefers CS. This is in line with a study done in urban Nigeria, Iran mothers who had no knowledge about CD (OR = 1.6) times more likely preferred CD [36, 40] . This may be due pregnant mothers with lack of knowledge couldn't fully appreciate the health risks of maternal and fetal complications of CD.

# Conclusion

In this study the preference of cesarean delivery was high as compared to world health organization recommendation. A significant number of mothers involved in the study preferred C/S as their mode of delivery, previous Satisfaction on intra partum care, current pregnancy related obstetric problem, Knowledge of the Respondents towards caesarean delivery, Previous spontaneous abortion, Residence were significantly associated with Preference of caesarean delivery.

**Commented [CP27]:** Indicate the percentage instead

**Commented [CP28]:** Which kind of residence

## Recommendation

- **Health care providers**: should be council pregnant mothers about spontaneous abortion to reduce complication for the future pregnancy and for minimize preference of CD.
- Nurses, Midwives and other stakeholders in obstetric care should give health education and proper counselling during antenatal care to women on Caesarean section as well as birth preparedness and complication readiness.
- **MOH**: Designing strategies to enhance maternal satisfaction by strengthening adherence to intra partum care.
- **Researchers:** A qualitative study is also required to better understand women's perspectives toward preference of cesarean delivery especially among mothers who had a pregnancy-related complication.

## Strength and Limitations of the Study

- The main strength of this study is that try to incorporate variables that were not in previous studies. Like previous satisfaction on intra partum care, knowledge towards cesarean delivery.
- Recall bias; as a limitation.

**Commented [CP29]:** Counsel on **abortion?**

**Commented [CP30]:** Recall on what? Justify

## Consent for publication

Not applicable

## Availability of data and materials

Data will be available upon request from the corresponding author.

**Competing interests**

The authors declare that they have no competing interests.

**Funding**:

No funding was obtained for this study**.**

**Acknowledgment**

The authors would like to thank and sincerely appreciate the study participants, data collectors, supervisors, and Debre-Berhan city health Administrative.

# Author's contributions

**Conceptualization:** Lemlem Zewudu
**Data curation:** Lemlem Zewudu
**Formal analysis:** Lemlem Zewudu
**Investigation:** Lemlem Zewudu , Fetene Keshaun, Mulualem Silesh, Eyob Ketema Bogale, Aberham Demis and Mitiku Tefera
**Methodology:** Lemlem Zewudu , Fetene Keshaun, Mulualem Silesh, Eyob Ketema Bogale, Aberham Demis and Mitiku Tefera
**Project administration:** Lemlem Zewudu , Fetene Keshaun, Mulualem Silesh, Eyob Ketema Bogale, Aberham Demis and Mitiku Tefera
**Resources:** Lemlem Zewudu , Fetene Keshaun, Mulualem Silesh, Eyob Ketema Bogale, Aberham Demis and Mitiku Tefera
**Software:** Lemlem Zewudu , Fetene Keshaun, Mulualem Silesh, Eyob Ketema Bogale, Aberham Demis and Mitiku Tefera
**Supervision:** Lemlem Zewudu , Fetene Keshaun, Mulualem Silesh, Eyob Ketema Bogale, Aberham Demis and Mitiku Tefera
**Validation:** Lemlem Zewudu , Fetene Keshaun, Mulualem Silesh, Eyob Ketema Bogale, Aberham Demis and Mitiku Tefera

**Visualization:** Lemlem Zewudu , Fetene Keshaun, Mulualem Silesh, Eyob Ketema Bogale, Aberham Demis and Mitiku Tefera

**Writing – original draft:** Lemlem Zewudu , Fetene Keshaun, Mulualem Silesh, Eyob Ketema Bogale, Aberham Demis and Mitiku Tefera

**Writing – review & editing:** Lemlem Zewudu , Fetene Keshaun, Mulualem Silesh, Eyob Ketema Bogale, Aberham Demis and Mitiku Tefera

# Reference


1. Jalali, N., et al., *Pregnant women's views on the type of delivery: determinants and relationships.* پرستاري و مامايي جامع نگر= Journal of Holistic Nursing and Midwifery, 2019. **3**(2588-3712).
2. Belizán, J.M., F. Althabe, and M.L. Cafferata, *Health consequences of the increasing caesarean section rates.* Epidemiology, 2007. **18**(4): p. 485-486.
3. Hobbs, A.J., et al., *The impact of caesarean section on breastfeeding initiation, duration and difficulties in the first four months postpartum.* BMC pregnancy and childbirth, 2016. **16**(1): p. 1-9.
4. Omar, A.A.A. and S.H.A. Anza, *Frequency Rate and Indications of Caesarean Sections at Prince Zaid Bin Al Hussein Hospital-Jordan.* JRMS, 2012. **19**(1): p. 82-6.
5. Barry, M.J. and S. Edgman-Levitan, *Shared decision making—The pinnacle patient-centered care.* 2012.
6. Dean, S.V., et al., *Preconception care: nutritional risks and interventions.* Reproductive health, 2014. **11**(3): p. 1-15.
7. Chelangat, C., M. Kipmerewo, and B. Mukabana, *Factors Influencing Women's Preferred Mode of Delivery in Kericho County Hospitals, Kenya.* Global Journal of Health Science, 2021. **13**(11): p. 89.
8. Health, N.I.o., *State-of-the-science conference statement: Cesarean delivery on maternal request.* Obstet Gynecol, 2006. **107**: p. 1386-1397.
9. Miller, S., et al., *Beyond too little, too late and too much, too soon: a pathway towards evidence-based, respectful maternity care worldwide.* The Lancet, 2016. **388**(10056): p. 2176-2192.
10. Eyowas Abebe, F., et al., *Factors leading to cesarean section delivery at Felegehiwot referral hospital, Northwest Ethiopia: a retrospective record review.* Reproductive Health, 2016.
11. Yingwani-Mlambo, L. and M. Mabenge. *Analysis of caesarean sections using Robson 10–group classification system in a tertiary hospital, Port Elizabeth*. in *Obstetrics and Gynaecology Forum*. 2020. In House Publications.
12. Betrán, A.P., et al., *The increasing trend in caesarean section rates: global, regional and national estimates: 1990-2014.* PloS one, 2016. **11**(2): p. e0148343.
13. Dhodapkar, S.B., et al., *Analysis of caesarean sections according to Robson's ten group classification system at a tertiary care teaching hospital in South India.* Int J Reprod Contracept Obstet Gynecol, 2015. **4**(3): p. 745-9.
14. Makhanya, V., L. Govender, and J. Moodley, *Utility of the Robson Ten Group Classification System to determine appropriateness of caesarean section at a rural regional hospital in KwaZulu-Natal, South Africa.* South African Medical Journal, 2015. **105**(4): p. 292-295.

15. Mose, A. and H. Abebe, *Magnitude and associated factors of caesarean section deliveries among women who gave birth in Southwest Ethiopia: institutional-based cross-sectional study.* Archives of Public Health, 2021. **79**: p. 1-9.
16. Abubeker, F.A., et al., *Analysis of cesarean section rates using Robson ten group classification system in a tertiary teaching hospital, Addis Ababa, Ethiopia: a cross-sectional study.* BMC Pregnancy and Childbirth, 2020. **20**: p. 1-7.
17. Alemu, A.A. and L.B. Zeleke, *Magnitude and Determinants of Primary Cesarean Section Among Women Who Gave Birth in Shire, Northern Ethiopia.* Open Access Surgery, 2020. **13**: p. 53-59.
18. Csa, I., *Central statistical agency (CSA)[Ethiopia] and ICF.* Ethiopia demographic and health survey, Addis Ababa, Ethiopia and Calverton, Maryland, USA, 2016. **1**.
19. Tegegne, T.K., et al., *Spatial and hierarchical Bayesian analysis to identify factors associated with caesarean delivery use in Ethiopia: Evidence from national population and health facility data.* Plos one, 2022. **17**(11): p. e0277885.
20. Gebremedhin, S., *Trend and socio-demographic differentials of Caesarean section rate in Addis Ababa, Ethiopia: analysis based on Ethiopia demographic and health surveys data.* Reproductive health, 2014. **11**(1): p. 1-6.
21. Welay, F.T., et al., *Delivery mode preference and associated factors among pregnant mothers in Harar regional state, Eastern Ethiopia: a cross-sectional study.* BioMed Research International, 2021. **2021**.
22. Ryding, E.L., et al., *Pregnant women's preference for cesarean section and subsequent mode of birth–a six-country cohort study.* Journal of Psychosomatic Obstetrics & Gynecology, 2016. **37**(3): p. 75-83.
23. Løvåsmoen, E.M.L., et al., *Women's preference for caesarean section and the actual mode of delivery–comparing five sites in Norway.* Sexual & reproductive healthcare, 2018. **16**: p. 206-212.
24. Tenaw, Z., et al., *Maternal preference, mode of delivery and associated factors among women who gave birth at public and private hospitals in Hawassa City, Southern Ethiopia.* Annals of Global Health, 2019. **85**(1).
25. Eide, K.T. and K. Bærøe, *How to reach trustworthy decisions for caesarean sections on maternal request: a call for beneficial power.* Journal of Medical Ethics, 2021. **47**(12): p. e45-e45.
26. Ghotbi, F., et al., *Women's knowledge and attitude towards mode of delivery and frequency of cesarean section on mother's request in six public and private hospitals in T ehran, I ran, 2012.* Journal of Obstetrics and Gynaecology Research, 2014. **40**(5): p. 1257-1266.
27. Misaeli, C., et al., *Factors associated with women's intention to request caesarean delivery in Dar es Salaam, Tanzania.* South African Journal of Obstetrics and Gynaecology, 2017. **23**(2): p. 56-62.
28. Joshi, A., M. Thapa, and O.B. Panta, *Maternal attitude and knowledge towards modes of delivery.* Journal of Nepal Health Research Council, 2018. **16**(2): p. 209-214.
29. Karoni, H.F., et al., *Maternal satisfaction among vaginal and cesarean section delivery care services in Bahir Dar city health facilities, Northwest Ethiopia: a facility-based comparative cross-sectional study.* BMC pregnancy and childbirth, 2020. **20**(1): p. 1-14.
30. Yusuf, A., et al., *PREVALENCE OF CESARIAN DELIVERY AND ASSOCIATED FACTORS AMONG MOTHERS WHO GAVE BIRTH AT PUBLIC HOSPITALS, JIGJIGA, ETHIOPIA.* 2023.
31. Zewude, B., G. Siraw, and Y. Adem, *The Preferences of Modes of Child Delivery and Associated Factors Among Pregnant Women in Southern Ethiopia.* Pragmatic and Observational Research, 2022: p. 59-73.
32. Mohamed, S.L., H. Fouly, and M. Mohamed, *Comparative study between Assiut and Zagazig regarding women preferences for mode of delivery.* IOSR Journal of Nursing and Health Science (IOSR-JNHS), 2016. **5**(3): p. 35-45.

33. Kidane, A., et al., *Maternal satisfaction on delivery care services and associated factors at public hospitals in eastern Ethiopia.* International Health, 2023. **15**(2): p. 189-197.
34. Venugopala, K., V. Rashmi, and B. Odhav, *BioMed Res. Intl.* Article ID, 2013. **963248**: p. 14.
35. Tesfaye, R., et al., *Client satisfaction with delivery care service and associated factors in the public health facilities of Gamo Gofa zone, Southwest Ethiopia: in a resource limited setting.* Obstetrics and gynecology international, 2016. **2016**.
36. Maharlouei, N., et al., *The preference of Iranian women to have normal vaginal or cesarean deliveries.* Journal of research in medical sciences: the official journal of Isfahan University of Medical Sciences, 2013. **18**(11): p. 943.
37. Neuman, M., et al., *Prevalence and determinants of caesarean section in private and public health facilities in underserved South Asian communities: cross-sectional analysis of data from Bangladesh, India and Nepal.* BMJ open, 2014. **4**(12): p. e005982.
38. Parvej, M.I., M. Tabassum, and N. Aktar, *Preferences between Caesarean Section and Normal Vaginal Delivery among the reproductive women in Bangladesh.* Journal of Applied Science, Engineering, Technology, and Education, 2021. **3**(1): p. 82-89.
39. Adu-Bonsaffoh, K., E. Tamma, and J. Seffah, *Preferred mode of childbirth among women attending antenatal clinic at a tertiary hospital in Ghana: a cross-sectional study.* African Health Sciences, 2022. **22**(2): p. 480-488.
40. Aziken, M., L. Omo-Aghoja, and F. Okonofua, *Perceptions and attitudes of pregnant women towards caesarean section in urban Nigeria.* Acta obstetricia et gynecologica Scandinavica, 2007. **86**(1): p. 42-47.
