# Supplementary material for: Preference of cesarean delivery and its associated factors among pregnant women attending ante natal care at public health facilities of Debrebrehan City, Ethiopia: Cross-sectional study
Source: PLoS One. 2024 Jan 31;19(1):e0296990. doi: 10.1371/journal.pone.0296990 (PMC10829985; doi:10.1371/journal.pone.0296990)
Supplement: Attachment — Submitted filename: Response to Reviewers.docx [file pone.0296990.s006.docx]

**RESPONSE TO EDITOR AND REVIEWERS**

**RESPONSE TO EDITOR**

RESPONSE: Thank you for coordinating the review process and fruitful comments. We have revised the manuscript and addressed Reviewer’s comments.

Journal requirements: When submitting your revision, we need you to address these additional requirements.

**JOURNAL REQUIREMENTS:** 1. Please ensure that your manuscript meets PLOS ONE's style requirements, including those for file naming.

The PLOS ONE style templates can be found at https://journals.plos.org/plosone/s/file?id=wjVg/PLOSOne_formatting_sample_main_body.pdf and https://journals.plos.org/plosone/s/file?id=ba62/PLOSOne_formatting_sample_title_authors_affiliations.pdf

**RESPONSE: Thank you for your kind words and fruitful comments. We have ensured for you that our manuscript meets PLOS ONE's style requirements, including those for file naming.**

**JOURNAL REQUIREMENTS:** 2. We noticed you have some minor occurrence of overlapping text with the following previous publication(s), which needs to be addressed:

- https://doi.org/10.1155/2021/1751578

- https://doi.org/10.2147/RMHP.S330932

- https://doi.org/10.5539/gjhs.v13n11p89

- ttps://dx.doi.org/10.4314/ahs.v22i2.56

In your revision ensure you cite all your sources (including your own works), and quote or rephrase any duplicated text outside the methods section. Further consideration is dependent on these concerns being addressed.

RESPONSE: Thank you for your kind words and fruitful comments. We have revised our manuscript based on the comments

**JOURNAL REQUIREMENTS:** 3. In your Data Availability statement, you have not specified where the minimal data set underlying the results described in your manuscript can be found. PLOS defines a study's minimal data set as the underlying data used to reach the conclusions drawn in the manuscript and any additional data required to replicate the reported study findings in their entirety. All PLOS journals require that the minimal data set be made fully available. For more information about our data policy, please see http://journals.plos.org/plosone/s/data-availability.

"Upon re-submitting your revised manuscript, please upload your study’s minimal underlying data set as either Supporting Information files or to a stable, public repository and include the relevant URLs, DOIs, or accession numbers within your revised cover letter. For a list of acceptable repositories, please see http://journals.plos.org/plosone/s/data-availability#loc-recommended-repositories. Any potentially identifying patient information must be fully anonymized.

Important: If there are ethical or legal restrictions to sharing your data publicly, please explain these restrictions in detail. Please see our guidelines for more information on what we consider unacceptable restrictions to publicly sharing data: http://journals.plos.org/plosone/s/data-availability#loc-unacceptable-data-access-restrictions. Note that it is not acceptable for the authors to be the sole named individuals responsible for ensuring data access.

We will update your Data Availability statement to reflect the information you provide in your cover letter.

**RESPONSE: Thank you for your kind words and fruitful comments. We have revised our Data Availability statement as: All datasets used and/or analyzed during the current study are available with in the manuscript.**

**JOURNAL REQUIREMENTS:** Please include a separate caption for each figure in your manuscript.

**RESPONSE: Thank you for your kind words and fruitful comments. We have revised it and we would like to notify for you that there is no figure in our manuscript.**

**JOURNAL REQUIREMENTS:** Please include captions for your Supporting Information files at the end of your manuscript, and update any in-text citations to match accordingly. Please see our Supporting Information guidelines for more information: <http://journals.plos.org/plosone/s/supporting-information.>

RESPONSE: Thank you for your kind words and fruitful comments. We have

**JOURNAL REQUIREMENTS:** Please review your reference list to ensure that it is complete and correct. If you have cited papers that have been retracted, please include the rationale for doing so in the manuscript text, or remove these references and replace them with relevant current references. Any changes to the reference list should be mentioned in the rebuttal letter that accompanies your revised manuscript. If you need to cite a retracted article, indicate the article’s retracted status in the References list and also include a citation and full reference for the retraction notice.

**RESPONSE: Thank you for your kind words and fruitful comments. We have reviewed our reference list and we ensured that it is complete and correct.**

**RESPONSE TO REVIEWER 1:**

RESPONSE: We thank the reviewer for kind words and fruitful comments. We have revised our manuscript based on the comments as described below.

**REVIEWER COMMENT:** Reviewer #1: 1. Stages of pregnancy might be considered because the intention for preferring mode of delivery may be different in the 1st, 2nd, and 3rd trimester of pregnancy. If a first-trimester pregnant mother were included in this study, the criteria for diagnosing a nonviable pregnancy should be included.

**RESPONSE: We thank the reviewer for kind words and fruitful comments. We have no data related with trimester of pregnancy, instead we prefer to collect data based on ANC followup visit. This may predict their trimester of pregnancy. In this study we did not exclude first-trimester pregnant mother and the criteria for diagnosing a nonviable pregnancy was urine HCG.**

**REVIEWER COMMENT: 2.** The tables in this manuscript are not standardized tables. The tables in the manuscript need formatting for readability.

**RESPONSE: We thank the reviewer for kind words and fruitful comments. We have revised the tables in the manuscript formatting as standardized to increase readability.**

**REVIEWER COMMENT: 3.** The manuscript needs language editing, especially grammatical errors, which should be corrected by consulting a language expert or using a grammar checker.

**RESPONSE:** We thank the reviewer for kind words and fruitful comments. We have revised it by editing to fix grammatical errors.

**REVIEWER COMMENT: 4.** There is no coherence between the result, conclusion, and recommendations. Unrelated recommendations are written in this manuscript.

**RESPONSE: We thank the reviewer for kind words and fruitful comments. We have revised it to follow coherence between the result, conclusion, and recommendations. We also revised our recommendation section by replacing unrelated recommendations with recommendations based on our findings.**

**RESPONSE TO REVIEWER 2:**

**REVIEWER COMMENT:**  Reviewer #2: 1. Exclusion criteria: whay were the mother who had cesearean section prior excluded? please explain why they were excluded

**RESPONSE: We thank the reviewer for kind words and fruitful comments. We would like to notify for you that the mother who had previous cesearean section might have cesarean section indication for the next pregnancy and this might be told for them during post operation hospital stay. We kindly ask you to consider this during review.**

**REVIEWER COMMENT: 2.** please make the table of bivariate and multivariate in the results section, then explain how the significant factors would explain the findings

**RESPONSE: We thank the reviewer for kind words and fruitful comments. We can make sure for you that table of bivariate and multivariate in the results section placed above discussion in the result section and we explained significant factors associated with the outcome variable.**

**REVIEWER COMMENT: 3.** get help with editing the essay part of the English before sending it for publication, for better understanding

**RESPONSE:** We thank the reviewer for kind words and fruitful comments. We have revised it by editing to fix grammatical errors.

**REVIEWER COMMENT: 4.** Provide a point on the recomendation that adresses the concerns of clinicians

**RESPONSE: We thank the reviewer for kind words and fruitful comments. We have revised it based on your comment as: Clinicians who are working on delivery room should better to improve their service provision by using patient centered care to increase their patient satisfaction.**

**REVIEWER COMMENT:** 5. provide recomendatoions guided by the WHO guidelines and the Ministry of Health

**RESPONSE: We thank the reviewer for kind words and fruitful comments. We have revised it based on your comment to fix it.**

**RESPONSE TO REVIEWER 3**:

**REVIEWER COMMENT: -** Work on the background comments on the manuscript

**RESPONSE: We thank the reviewer for kind words and fruitful comments. We have revised our manuscript based on the comments.**
**REVIEWER COMMENT: -** Work on the ethical considerations as presented including using names of health facilities assessed

**RESPONSE: We thank the reviewer for kind words and fruitful comments. We have ensured for you that the leader of listed facility manager gave their consent to included facility name in the study including publication.**
**REVIEWER COMMENT: -** Also look at the methodology to tweak a few areas

**RESPONSE: We thank the reviewer for kind words and fruitful comments. We have revised our methodology based on the comments.**

**REVIEWER COMMENT: -** Work on the results and discussions attached in the manuscript reviewed attached

**RESPONSE: We thank the reviewer for kind words and fruitful comments. We have revised our results and discussions section as per your comments which attached in the manuscript.**
