# Supplementary material for: Preference of cesarean delivery and its associated factors among pregnant women attending ante natal care at public health facilities of Debrebrehan City, Ethiopia: Cross-sectional study
Source: PLoS One. 2024 Jan 31;19(1):e0296990. doi: 10.1371/journal.pone.0296990 (PMC10829985; doi:10.1371/journal.pone.0296990)
Supplement: Attachment — Submitted filename: PONE-D-23-28572_R1Manscripte Second Review.docx [file pone.0296990.s007.docx]

**Preference of Cesarean Delivery and Its Associated Factors Among Pregnant Women Attending Ante Natal Care at Public Health Facilities of Debrebrehan City, Ethiopia: Cross-Sectional Study**

**Lemlem Zewudu^1^, Fetene Keshaun^2^, Mulualem Silesh^2^ , Mitiku Tefera^2^*, Eyob Ketema Bogale^3^ , Aberham Demis ^4^ , Zewedie Yeshaw Tekle^5^**

^1^Debre Berhan compressive specialized Hospital Amhara region, Debre Berhan, Ethiopia.

^2^Department of Midwifery School of Nursing and Midwifery, Asrat woldeyes health science campus, Debre Berhan University, Ethiopia.

^3^Health Promotion and Behavioral Sciences Department, School of public health, college of medicine and health sciences, Bahir Dar University, Bahir Dar, Ethiopia

^4^ Department of Midwifery, Deber-Berehan Health Science College, Debre Berhan, Amhara, Ethiopia.

^5^Department of Midwifery, Deber-Berehan Health center, Debre Berhan, Amhara, Ethiopia.

***** Corresponding Author: **Mitiku Tefera**

# **ABSTRACT**

**Background**: A Caesarean section is a surgical procedure used to prevent or treat life-threatening maternal or fetal complications. Women's delivery preferences have become a global issue of interest to many researchers and clinicians, especially given the ever-increasing rate of cesarean sections. There is limited data on the preference for cesarean delivery and its associated factors for Ethiopian women, particularly in the study area. The aim of the study is to assess the preference for cesarean delivery and its associated factors among pregnant women attending antenatal care at public health facilities in Debre Berhan, Ethiopia, in 2023.

**Methods**: An institution-based cross-sectional study design was done from May 5–20, 2023, among 512 participants, and a multi-stage sampling technique was used. The data were collected by using interviewer-administered semi-structured questionnaires. The data were entered by Epi Data version 4.6 and then transferred to SPSS version 25 for analysis. With logistic regression, those variables with a p-value <0.25 in the bivariate analysis were candidates for multivariate logistic regression, and variables with a p-value <0.05 were considered statistically significant.

**Result:** The preference for a cesarean section was 26%, with a CI of 22.3% to 29.9%. Pregnant mothers who were not satisfied with their previous intrapartum care (AOR; 6.3 CI=(3.5-11), P=0.01), had no knowledge about cesarean delivery (AOR; 2.9; 95% CI=1.6-5.3), P=0.01), had a previous history of spontaneous abortion (AOR; 3.1; 95% CI=(1.5-6.3), P=0.001), lived in an urban area (AOR; 1.9; 95% CI=(1.0-3.5), P=0.038), and had a current pregnancy-related problem (AOR; 4.8; 95% CI=1.9-10), P=0.001) were significantly associated with the preference for cesarean delivery.

**Conclusion:** In this study, the preference for cesarean delivery was high as compared to the World Health Organization recommendation. Pregnant mothers who were not satisfied with their previous intrapartum care, had no knowledge about cesarean delivery, had a previous history of spontaneous abortion, had an urban residence, and had a current pregnancy-related problem were significantly associated with a preference for caesarean delivery. Clinicians who are working in the delivery room should improve their service provision by using patient-centered care to increase patient satisfaction.

**Key words:** Preference of cesarean delivery, Pregnant Women, Ante Natal Care, Public Health Facilities, Ethiopia

# **Introduction**

A Caesarean section is a surgical procedure used to prevent or treat life-threatening maternal or fetal complications [1]. Pregnant women are normally involved in the decision-making process concerning the mode of delivery, and many factors affect their decision. These processes are influenced by a person’s environment, values, personality, knowledge, and insight, which influence each other interactively [2].

Maternity delivery is one of the most important healthcare services in all countries [3]. The preference for cesarean delivery is defined as choosing a cesarean section as a mode of delivery [4]. Women’s preferences for mode of delivery have emerged as a global subject of interest to many researchers and clinicians, especially with the steady increase in the rate of cesarean sections (CS), even though the World Health Organization (WHO) advises a maximum of 10-15% acceptable rate of cesarean sections [5]. Some data show cesarean section rates above 15% are not linked to further declines in maternal and neonatal mortality and morbidity [6].

The right to prefer a mode of delivery is a crucial component of compassionate and respectful care in modern obstetrics, as it fosters both maternal and neonatal well-being [7].

Cesarean delivery at the mother's request (CDMR) is a branch of elective cesarean sections performed not according to medical indication but at the mother's request [8].

C-sections have become a prominent indicator of progress in emergency obstetric care and a method to avert complications during labor and delivery[9]. Modern obstetric practice has seen increases in primary CS rates everywhere for medical, social, economic, and legal reasons [10]. For instance, studies have shown that Port Elizabeth has the highest CS rates (55.6%)[11],  in Latin America (40.5%) [12] in the southern India region (32%)[13], in South Africa the CS rate is (42.4%),[14], and in Ethiopia also the CS rate is between 20.2 and 38.3% of mothers who have undergone cesarean section [15-17].

In Ethiopia, between 2000 and 2016, there was a slight increase in the national cesarean section rate from 0.7% in 2000 to 1.9% in 2016 [18]. Based on various attributes, differences continued to exist. Compared to rural areas, which had a cesarean section rate of 0.9%, urban areas had a cesarean section rate of 10.6% in 2016 [18-20]. Determining their preferences in terms of mode of delivery will help reduce maternal and prenatal morbidity and mortality (19).

The advancement of delivery care, including cesarean delivery, has greatly improved the outcomes of birth globally, with a significant reduction in maternal morbidity and mortality. However, the evidence to support this is limited in Africa, especially Ethiopia, and more particularly in the study area. The study aims at assessing the factors influencing women’s preference for the cesarean section mode of delivery.

# **Methods and Materials**

**Study Design and Settings**

An institution-based cross-sectional study design was conducted in Debre Berhan town public health facilities from May 5 to May 20, 2023. Debre Berhan is located 130 km from Addis Ababa and 695 km from Bihar Dar, the capital city of Amhara Regional State. The total population of Debre Berhan town is 202,226; of the total population, 106,388 are females and 6,815 are pregnant mothers. According to the zonal health department report, Debre Berhan town has ten public health facilities, which are 2 government hospitals (of which are Debre Berhan comprehensive specialized hospital and Hakim Gizaw Hospital) and 8 health centers, which are Debre Berhan health center, Tebase health center, Ayer tena health center, Chacha health center, keyt health centers, Goshebado health center, Debre, and Enkulal Koso health center.

**Source of population:** all pregnant mothers who attended their ANC at a public health institution in Debre Berhan city.

**Study population:** all pregnant mothers who attended their ANC at selected health institutions in Debre Berhan City.

**Inclusion Criteria:** All pregnant women who had one or more deliveries and who attended the ANC during the data collection period. In this study, the criteria for diagnosing a nonviable pregnancy for a first-trimester pregnant mother was urine HCG.

**Exclusion criteria:** pregnant mothers who had previous CS scars.

## **Sample size determination**

A single population proportion formula is used to calculate the sample size by considering the following statistical assumptions: P = proportion of the preference rate of CS among pregnant mothers from another study, 28.9%, Harar Regional State, Eastern Ethiopia[[21](https://mail.google.com/mail/u/0/#_ENREF_21)].

(Z α/2 = Z score of 95% CI, d= Margin of error (5%).

n = (1.96)2 *0.289*0.711/ (0.05)2 = 316* 1.5=474 Then after adding 10 % non-response rate, the sample size was 522.

## **Sampling technique and procedure**

A multi-stage sampling technique was used to select a representative sample. There are 10 public health facilities in Debre Berhan city; of these, 5 were selected using a simple random sampling method. The selected health facilities are Debere-Berhan Comprehensive Specialized Hospital (DBCSH), Debre Berhan Health Center, Tebase Health Center, Chacha Health Center, and Keyt Health Center. The sample was allocated proportionally for each health facility, and the allocation was done by using the average monthly ANC follow-up, which was 2100. Study participants were selected using a systematic random sampling technique. First, determine the sampling interval (K) value by dividing the total number of pregnant women attending antenatal care during the study period by the total sample size, which gives 2.01 ≈ 2.

The probability allocation sampling technique was to select (nf x n)/N = (Sample final * number of total pregnant women attending antenatal care in each health facility/number of total pregnant women attending antenatal care within two weeks.

Where N is equal to 1050.

- Debre Berhan comprehensive specialized hospital = 522x 400/ 1050 = 198
- Debre Berhan Health Center = 522 x 220/ 1050 = 110
- Tebase Health Center = 522 x 150/1050 = 74
- Cacha Health Center = 522 x 176/ 1050= 88
- Keyt Health Center =522 x 164/1050= 81 (Fig 1)

Figure 1: Schematic representation of sampling procedure for determining the preference of C/S and its associated factors among pregnant mother attending ANC at public health facilities of Debre Birhan town, Ethiopia, 2023

## **Data collection methods**

Data was collected using semi-structured questionnaires adapted from a review of relevant literature [[22-26](https://mail.google.com/mail/u/0/#_ENREF_22)]. All questions were written in English and translated into Amharic (the local language) and then back to English by two different language experts to check for consistency and clarity. The questionnaire is divided into six sections (1–5) to obtain data on the socio-demographic characteristics of the respondents, obstetric factors, knowledge towards cesarean delivery, attitude towards cesarean delivery, and preference of cesarean delivery parts(see **S1**)

## **Variables of the study**

**Dependent variable: p**reference of cesarean delivery.

###### Independent variable: socio demographic factors, obstetric factors, knowledge towards cesarean delivery, attitude towards ceesarean delivery and maternal satisfaction on previous intra partum care.

## **Operational definitions**

# **Preference for cesarean delivery** implies the patient's choice of cesarean delivery without any fetal or maternal indication [27]. **Maternal Knowledge:** To assess maternal knowledge of cesarean delivery, the questions are adapted.1 point was given for each correct response, and 0 points for incorrect and ‘I don’t know’ answers. The overall maternal knowledge score was described as good (7–10), intermediate (4–6), and poor (0–3) [26]. **Attitude towards cesarean delivery** The questionnaire for attitude assessment was served in a Likert scale format with strongly agree (score 5), agree (score 4), neutral (score 3), disagree (score 2), and strongly disagree (score 1). Attitude toward CD was assessed with 10 statements for cesarean delivery. A median attitude score was computed for each respondent for all the statements to find the overall attitude of women towards that mode of delivery. A median attitude score of 3 or less was considered a negative attitude, and a score of more than 3 was considered a positive attitude toward that particular mode of delivery [28]. **Maternal satisfaction** is the satisfaction of mothers during service delivery. The level of satisfaction was assessed on a 5-point Likert scale (1, very dissatisfied; 2, dissatisfied; 3, neutral; 4, satisfied; 5, very satisfied).1 point was given to satisfied and 0 points to unsatisfied. Those who were satisfied with ≥75% of the items were categorized as satisfied (those who responded as very satisfied, satisfied, or neutral), and those who were satisfied with <75% of the items were categorized as ‘unsatisfied’ (those who responded dissatisfied or very dissatisfied [29]. **Data Quality Assurance** Five diploma midwives participated as data collectors, and one BSC midwife controlled the overall activity of a data collection method as a supervisor. One day of training was given to data collectors on the objective of the study. Pre-testing on a similar set of respondents was done in Debre Sina Primary Hospital. It was done to check for reliability, appropriateness of format, and wording of the questionnaire. **Data processing and analysis** Following completion of the data collection, questionnaires were checked for completeness and consistency, and data was entered using Epi Data version 4.6, then transferred to SPSS version 25 for analysis. Binary and multiple logistic regression analyses were performed. Variables with a p-value of 0.25 in the bi-variable analysis were considered for the multivariate analysis to control the effect of confounding variables. The odds ratio, along with a 95% confidence interval (CI), was computed to ascertain the strength of the association between the explanatory and outcome variables. The regression model fitness was checked by the Hosmer-Lemshow goodness test (0.077) and Nagelkerke R square (0.463). The multi-collinearity assumption was checked by the variance inflation factor (VIF), and there is no multi-collinearity. **Ethical considerations** Informed written consent was obtained from each study subject after a clear explanation of the purpose of the study. An official letter of cooperation was obtained for each selected health facility from the Debere-Berhan University Asrat Woldeyes Health Science campus (protocol number IRB-135). We considered and agreed on the beneficence, no maleficence, and autonomy of the participants before beginning data collection by obtaining consent from all chief executive officers of the hospital and health center head. The purpose of the study was explained to the study participants, and confidentiality was ensured. At all levels, officials were contacted, and permission was secured.

# **Results**

## **Socio-Demographic Characteristics of Respondents**

A total of 512 participants were enrolled in this study, with a response rate of 98%. The age of the mothers ranges from 18 to 45 years old, with a mean age of 32.9 years. The marital status of the participants revealed that 84.4% of them were married during the period of data collection. Moreover, 30.5% of respondents reported having completed primary education (Table 1).

**Table1.Socio demographic characteristics of pregnant women attending ANC in selected Public health facilities in Debre Berhan, Ethiopia, 2023.**

| **Variables** | **Category** | **Frequency** | **Percentage (%)** |
| --- | --- | --- | --- |
| Residence | Urban | 337 | 65.8% |
|  | Rural | 175 | 34.2% |
| Age | 18-25 | 113 | 22% |
|  | 26-35 | 283 | 55.3% |
|  | 36-45 | 116 | 22.7% |
|  | Single | 37 | 7.2% |
| Marital status | Married | 431 | 84.2% |
|  | Divorced | 31 | 6.1% |
|  | Widowed | 13 | 2.5% |
| Income | >2500 | 145 | 28.3% |
|  | 2500-4000 | 120 | 23.4% |
|  | 4001-10000 | 135 | 26.4% |
|  | >10000 | 112 | 21.9% |
| Occupation | Employed | 166 | 32.4% |
|  | Un employed | 346 | 67.6% |
| Educational status | No formal education | 143 | 31.3% |
|  | Primary education | 131 | 28.7% |
|  | Secondary education | 67 | 14.7% |
|  | College and above | 11 | 25.4% |
| Partners Educational status | No formal education | 143 | 22.7% |
|  | Primary education | 131 | 13.1% |
|  | Secondary education | 67 | 25.6% |
|  | College and above | 116 | 13.1% |
| Occupational status | Employed | 174 | 34% |
|  | Un employed | 338 | 66% |

## **Obstetric characteristics of the respondents**

Among the 512 respondents, 81.6% were multi-para. Of that, 63% were delivered at a health center during the previous childbirth. The majority (70%) of the respondents had planned pregnancy; 15.8% of participants had previous pregnancy-related complications; and 6.1% had current pregnancy-related complications. On the other hand, 46.8% of respondents reported having a close friend or a family member who has been delivered through a cesarean section. One-fourth of participants were not satisfied with previous intrapartum care (Table 2).

**Table 2: Obstetric characteristics of pregnant women attending ANC in selected Public health facilities in Debre Berhan, Ethiopia, 2023**

| **Variables** | **Category** | **Frequency** | **Percentage (%)** |
| --- | --- | --- | --- |
| **Previous history of spontaneous abortion** | Yes | 78 | 15.2 |
|  | No | 434 | 48.8 |
| **Current number of ANC contact** | First Visit | 234 | 45.7 |
|  | Second Visit | 101 | 19.7 |
|  | Third Visit | 49 | 9.6 |
|  | 4th Visit | 46 | 9.0 |
|  | > 4th visit | 82 | 16.0 |
| **Parity** | Primi para | 94 | 18.4 |
|  | Multi | 418 | 81.6 |
| **Previous pregnancy related problem** | Yes | 66 | 12.3 |
|  | No | 449 | 87.7 |
| **Planed pregnancy** | Yes | 352 | 68.8 |
|  | No | 160 | 31.3 |
| **Current pregnancy related problem** | Yes | 36 | 7 |
|  | No | 476 | 93 |
| **Discussion with Partners** | Yes | 306 | 59.8 |
|  | No | 206 | 40.2 |
| **Partner’s support to preference** | Yes | 294 | 57.4 |
|  | No | 218 | 42.6 |
| **Previous intra partum care satisfaction** | Yes | 383 | 74.8 |
|  | No | 129 | 25.2 |
| **Attitude towards CD** | Positive attitude | 369 | 70.9 |
|  | Negative attitude | 149 | 29.1 |
| **Knowledge towards CD** | Not knowledgeable | 199 | 38.9 |
|  | Intermediate | 109 | 21.3 |
|  | knowledgeable | 204 | 39.8 |

## **Preference of Caesarean Delivery and their Reasons for it**

To determine women’s preference for cesarean delivery, 26%, with a CI of 22.3%–29.9%, of the respondents prefer cesarean delivery as a mode of delivery. Of the mothers who preferred cesarean section as the mode of delivery, about one-fourth of participants were due to CS having less labor pain than vaginal delivery (Fig 2),(Table 3).

Figure 2: Maternal preference of caesarean delivery among pregnant women attending ANC in selected Public health facilities in Debre Berhan, Ethiopia, 2023

**Table 3: Reasons behind Women’s Preference' for Caesareans delivery Among pregnant women attending ANC in selected Public health facilities in Debre Berhan, Ethiopia, 2023**

| **Variable** | **Frequency** | **Percentage** |
| --- | --- | --- |
| CS has less Labour pain | 99 | 24.8 |
| Avoidance of emergency cesarean section | 22 | 5.5 |
| Safer for women | 69 | 17.3 |
| Less risk of fetal distress | 41 | 10.3 |
| A chance to choose specific date | 9 | 2.3 |
| quick restoration for sexual activity | 12 | 3.0 |
| A fashion | 42 | 10.5 |
| Prior negative experience from vaginal delivery | 55 | 13.7 |
| Health care providers were not encouraging and reassuring during previous vaginal delivery | 10 | 2.5 |
| Fear or the need to avoid episiotomy | 31 | 7.8 |
| Other | 9 | 2.3 |

# **Factors associated with Preference of caesareans Delivery**

The bivariate analysis showed that the knowledge of the respondents towards CS, previous satisfaction during intrapartum care, residence, marital status, occupation, planned pregnancy, previous spontaneous abortion, current pregnancy-related obstetric problem, and discussion with partners about the mode of delivery were factors associated with the preference of cesarean delivery (p-value less than 0.25) and added to the multivariable logistic regression analysis. In multivariate logistic regressions, previous satisfaction with intrapartum care, current pregnancy-related obstetric problems, knowledge of the respondents towards cesarean delivery, previous spontaneous abortion, and residence were significantly associated with preference for cesarean delivery (p-value less than 0.05).

The result showed that pregnant women who lived in urban residences were 1.9 times more likely to prefer CS as compared with women who lived in rural. (AOR=1.9(1.03-3.5) P=0.038*). Pregnant women who had previous abortions were 3 times more likely to prefer CS compared to pregnant women who had no previous spontaneous abortion(AOR=3.1(1.5-6.3) P=0.001*).

Pregnant women dissatisfied with previous intrapartum care preferred CS for the current pregnancy as a mode of delivery, the degree of preference increased 6 times as compared to women who were satisfied. (AOR=6.3(3.58-11.29) P=0.01*

The other variable that was found to have a significant association was the knowledge of respondents about cesarean delivery, pregnant women who had no knowledge about cesarean delivery were 2.9 times more likely to prefer CS as compared to had knowledge about cesarean delivery (AOR= 2.9(1.6-5.3)P=0.01*). Pregnant mothers who have current pregnancy-related obstetric problems are 4.8 times more likely to prefer CS as compared to mothers who haven’t.(AOR=4.8, CI=(1.9-10),P=0.001)(Table 4).

**Table 4: Factors associated with Preference of caesarean delivery Caesareans Delivery Among pregnant women attending ANC in selected Public health facilities in Debre Berhan, Ethiopia, 2023**

| **Variables** | **Category** | **Preference of cesarean deliver** | | **COR (95%CI)** | **AOR(95% CI)** | **P value** |
| --- | --- | --- | --- | --- | --- | --- |
|  |  | **Yes** | **No** |  |  |  |
| **Knowledge of the respondents** | Not Knowledgeable | 66 | 133 | 2.2(1.4-3.5) | 2.9(1.6-5.3) | 0.001* |
|  | Intermediate | 30 | 79 | 1.71(0.9-2.9) | 2.4(0.22-5.0) | 0.312 |
|  | Knowledgeable | 37 | 167 | 1 | 1 |  |
| **Previous intra partum Satisfaction** | Satisfied | 73 | 56 | 7 (4.5-10) | 6.3(3.5-11) | 0.001* |
|  | Not Satisfied | 60 | 323 | 1 | 1 |  |
| **Residence** | Urban | 105 | 232 | 2.3(1.4-3.7) | 1.9(1.0-3.5) | 0.038* |
|  | Rural | 28 | 147 | 1 | 1 |  |
| **Occupation** | Employed | 64 | 102 | 2.5(1.6-3.7) | 0.5(0.26-1.07) | 0.77 |
|  | Non Employed | 69 | 277 | 1 | 1 |  |
| **Planed pregnancy** | Yes | 110 | 242 | 2.7(1.6-4.4) | 1.07(0.4-2.3) | 0.858 |
|  | No | 23 | 137 | 1 | 1 |  |
| **Previous history of spontaneous abortion** | Yes | 48 | 30 | 6.56(3.9-10.9) | 3.1(1.5-6.36) | 0.001* |
|  | No | 85 | 349 | 1 | 1 |  |
| **Maternal Education** | No formal Education | 19 | 135 | 0.1(0.6-0.21) | 0.10(0.03-1.02) | 0.112 |
|  | Primary Education | 21 | 135 | 0.13(0.69-0.23) | 0.1(0.46-1.2) | 0.431 |
|  | Secondary Education | 36 | 63 | 0.4(0.2-0.8) | 0.4(0.2-1.3) | 0.325 |
|  | College and above | 57 | 46 | 1 | 1 |  |
| **Current Pregnancy related problem** | Yes | 25 | 11 | 7.7(3.6-16) | 4.8(1.9-10) | 0.001* |
|  | No | 108 | 368 | 1 | 1 |  |
| **Discussion with partner** | Yes | 88 | 218 | 1.4(1.9-2.1) | 0.8(0.4-1.4) | 0.545 |
|  | NO | 45 | 161 | 1 | 1 |  |

# **Discussion**

Women's delivery preference is a subject that is widely researched and debated in many parts of the world. Women's autonomy, their satisfaction with childbirth, and their active participation in the decision-making process regarding the way they want to give birth to their children are becoming increasingly important. In Ethiopia, very little is known about women's preferences for delivery methods, and there is no evidence of mothers' preference for cesarean sections. Although CS rates in Ethiopia have also increased [[30](https://mail.google.com/mail/u/0/#_ENREF_30)]. The purpose of this study was to assess the preference for cesarean delivery and its associated factors among pregnant women attending antenatal care at public health facilities to fill the aforementioned gap.

In this study, the prevalence of cesarean delivery among pregnant women attending ANC in public health facilities was 26%. Similar studies have been conducted in Ethiopia and other countries. This result is almost similar to the study in the southern part of Ethiopia and in Harer, which is 24.6% and 28%, respectively [[21](https://mail.google.com/mail/u/0/#_ENREF_21), [31](https://mail.google.com/mail/u/0/#_ENREF_31)]. The similarity may be that both studies were conducted during the ANC visit. However, this study result showed a higher preference for CD compared to other studies conducted at the University Hospital of Asyut, Egypt, in six European countries (Belgium, Iceland, Denmark, Estonia, Norway, and Sweden), where the preference for C/S was 12.2% [[22](https://mail.google.com/mail/u/0/#_ENREF_22), [32](https://mail.google.com/mail/u/0/#_ENREF_32)]. The discrepancy emerged when this study interviewed mothers in the ANC unit while the reference studies were conducted in delivery units, which may reduce the tendency of mothers to choose CD because of fear of childbirth.

In this study, dissatisfaction with intrapartum care was significantly associated with a preference for cesarean delivery. This was in line with studies conducted in Debre Markos, Gamo Gofa Zone, and southwest Ethiopia [[33-35](https://mail.google.com/mail/u/0/#_ENREF_33)]. This could be because those who gave birth through spontaneous vaginal delivery may be experiencing labor pains. However, for those who have been delivered by CS, maybe the anesthesia relieves the pain of labor, and the surgery results in satisfaction.

Further, according to the findings, mothers who had previous spontaneous abortions were found to have a statistically significant relationship with their preference for CD. Mothers who had previous spontaneous abortions were three times more likely to prefer CD than mothers who hadn’t had spontaneous abortions. This result was slightly similar to another study that was conducted in Iran( OR =1.7) [[36](https://mail.google.com/mail/u/0/#_ENREF_36)].

There is also an association between preference for cesarean delivery and residence. The findings showed that living in an urban settlement was significantly associated with the preference for CD. Respondents living in urban areas had higher odds for the preference of CD compared to rural dwellers (odd ratio of 1.9). This is in line with other studies conducted in Ghana, Nepal, and Bangalore [[37-39](https://mail.google.com/mail/u/0/#_ENREF_37)]. These could be urban women who are more likely to be educated and hear about the CD and are also financially able to afford the increased costs of a CD. In addition, living in an urban settlement also improves access to quality medical facilities that are well-equipped to perform CS.

Pregnant mothers who have a current pregnancy-related obstetric problem were four times more likely to prefer CS as compared to mothers who didn’t. This finding is almost similar to a study conducted in Hawassa [[24](https://mail.google.com/mail/u/0/#_ENREF_24)]. This might be due to a fear of intrapartum complications.

Pregnant mothers who had no knowledge of cesarean delivery were two times more likely to prefer CS. This is in line with a study done in urban Nigeria and Iran, where mothers who had no knowledge preferred CD [[36](https://mail.google.com/mail/u/0/#_ENREF_36), [40](https://mail.google.com/mail/u/0/#_ENREF_40)]. This may be because pregnant mothers, with a lack of knowledge, couldn’t fully appreciate the health risks of maternal and fetal complications of CD.

# **Conclusion**

In this study, the preference for cesarean delivery was higher as compared to the World Health Organization recommendation. One-fourth of pregnant mothers involved in the study preferred C/S as their mode of delivery. A pregnant mother who was not satisfied with their previous intrapartum care had no knowledge about cesarean delivery, had a previous history of spontaneous abortion, had an urban residence, and had a current pregnancy-related problem were significantly associated with the preference for cesarean delivery.

# **Recommendation**

⮚⬝**Health care providers** should provide health education to increase the knowledge of pregnant mothers about cesarean delivery, including indications, and provide counsel about the relationship between previous history of spontaneous abortion and current pregnancy-related problems and their appropriate possible option of delivery.

⮚⬝Clinicians who are working in the delivery room should improve their service provision by using patient-centered care to increase patient satisfaction.

⮚⬝Nurses, midwives, and other stakeholders in obstetric care should give health education and proper counseling during antenatal care to women during cesarean sections, as well as birth preparedness and complication readiness.

⮚⬝**MOH**: designing strategies to enhance maternal satisfaction by strengthening adherence to intrapartum care.

⮚⬝**Researchers:** A qualitative study is also required to better understand women's perspectives toward the preference for cesarean delivery, especially among mothers who had a pregnancy-related complication.

# **Strength and Limitations of the Study**

- The main strength of this study is that try to incorporate variables that were not in previous studies. Like previous satisfaction on intra partum care, knowledge towards cesarean delivery.

**Consent for publication**

Not applicable

**Availability of data and materials**

All datasets used and/or analyzed during the current study are available with in the manuscript.

**Competing interests**

The authors declare that they have no competing interests.

**Funding**:

No funding was obtained for this study**.**

**Acknowledgment**

The authors would like to thank and sincerely appreciate the study participants, data collectors, supervisors, and Debre-Berhan city health Administrative.

Supporting Information

**S1 English version questioner**

S2 Letter of Ethical Consideration

**S3 Clinical study Checklist**

**S4 STROBE checklist**

**Author’s contributions**

**Conceptualization:** Lemlem Zewudu

**Data curation:** Lemlem Zewudu

**Formal analysis:** Lemlem Zewudu

**Investigation:** Lemlem Zewudu , Fetene Keshaun, Mulualem Silesh, Eyob Ketema Bogale, Aberham Demis and Mitiku Tefera

**Methodology:** Lemlem Zewudu , Fetene Keshaun, Mulualem Silesh, Eyob Ketema Bogale, Aberham Demis and Mitiku Tefera

**Project administration:** Lemlem Zewudu , Fetene Keshaun, Mulualem Silesh, Eyob Ketema Bogale, Aberham Demis and Mitiku Tefera

**Resources:** Lemlem Zewudu , Fetene Keshaun, Mulualem Silesh, Eyob Ketema Bogale, Aberham Demis and Mitiku Tefera

**Software:** Lemlem Zewudu , Fetene Keshaun, Mulualem Silesh, Eyob Ketema Bogale, Aberham Demis and Mitiku Tefera

**Supervision:** Lemlem Zewudu , Fetene Keshaun, Mulualem Silesh, Eyob Ketema Bogale, Aberham Demis and Mitiku Tefera

**Validation:** Lemlem Zewudu , Fetene Keshaun, Mulualem Silesh, Eyob Ketema Bogale, Aberham Demis and Mitiku Tefera

**Visualization:** Lemlem Zewudu , Fetene Keshaun, Mulualem Silesh, Eyob Ketema Bogale, Aberham Demis and Mitiku Tefera

**Writing – original draft:** Lemlem Zewudu , Fetene Keshaun, Mulualem Silesh, Eyob Ketema Bogale, Aberham Demis and Mitiku Tefera

**Writing – review & editing:** Lemlem Zewudu , Fetene Keshaun, Mulualem Silesh, Eyob Ketema Bogale, Aberham Demis and Mitiku Tefera

**Reference**

1.	Dean, S.V., et al., *Preconception care: nutritional risks and interventions.* Reproductive health, 2014. **11**(3): p. 1-15.

2.	Chelangat, C., M. Kipmerewo, and B. Mukabana, *Factors Influencing Women’s Preferred Mode of Delivery in Kericho County Hospitals, Kenya.* Global Journal of Health Science, 2021. **13**(11): p. 89.

3.	Jalali, N., et al., *Pregnant women’s views on the type of delivery: determinants and relationships.* پرستاري و مامايي جامع نگر= Journal of Holistic Nursing and Midwifery, 2019. **3**(2588-3712).

4.	Belizán, J.M., F. Althabe, and M.L. Cafferata, *Health consequences of the increasing caesarean section rates.* Epidemiology, 2007. **18**(4): p. 485-486.

5.	Hobbs, A.J., et al., *The impact of caesarean section on breastfeeding initiation, duration and difficulties in the first four months postpartum.* BMC pregnancy and childbirth, 2016. **16**(1): p. 1-9.

6.	Omar, A.A.A. and S.H.A. Anza, *Frequency Rate and Indications of Caesarean Sections at Prince Zaid Bin Al Hussein Hospital-Jordan.* JRMS, 2012. **19**(1): p. 82-6.

7.	Barry, M.J. and S. Edgman-Levitan, *Shared decision making—The pinnacle patient-centered care.* 2012.

8.	Health, N.I.o., *State-of-the-science conference statement: Cesarean delivery on maternal request.* Obstet Gynecol, 2006. **107**: p. 1386-1397.

9.	Miller, S., et al., *Beyond too little, too late and too much, too soon: a pathway towards evidence-based, respectful maternity care worldwide.* The Lancet, 2016. **388**(10056): p. 2176-2192.

10.	Eyowas Abebe, F., et al., *Factors leading to cesarean section delivery at Felegehiwot referral hospital, Northwest Ethiopia: a retrospective record review.* Reproductive Health, 2016.

11.	Yingwani-Mlambo, L. and M. Mabenge. *Analysis of caesarean sections using Robson 10–group classification system in a tertiary hospital, Port Elizabeth*. in *Obstetrics and Gynaecology Forum*. 2020. In House Publications.

12.	Betrán, A.P., et al., *The increasing trend in caesarean section rates: global, regional and national estimates: 1990-2014.* PloS one, 2016. **11**(2): p. e0148343.

13.	Dhodapkar, S.B., et al., *Analysis of caesarean sections according to Robson’s ten group classification system at a tertiary care teaching hospital in South India.* Int J Reprod Contracept Obstet Gynecol, 2015. **4**(3): p. 745-9.

14.	Makhanya, V., L. Govender, and J. Moodley, *Utility of the Robson Ten Group Classification System to determine appropriateness of caesarean section at a rural regional hospital in KwaZulu-Natal, South Africa.* South African Medical Journal, 2015. **105**(4): p. 292-295.

15.	Mose, A. and H. Abebe, *Magnitude and associated factors of caesarean section deliveries among women who gave birth in Southwest Ethiopia: institutional-based cross-sectional study.* Archives of Public Health, 2021. **79**: p. 1-9.

16.	Abubeker, F.A., et al., *Analysis of cesarean section rates using Robson ten group classification system in a tertiary teaching hospital, Addis Ababa, Ethiopia: a cross-sectional study.* BMC Pregnancy and Childbirth, 2020. **20**: p. 1-7.

17.	Alemu, A.A. and L.B. Zeleke, *Magnitude and Determinants of Primary Cesarean Section Among Women Who Gave Birth in Shire, Northern Ethiopia.* Open Access Surgery, 2020. **13**: p. 53-59.

18.	Csa, I., *Central statistical agency (CSA)[Ethiopia] and ICF.* Ethiopia demographic and health survey, Addis Ababa, Ethiopia and Calverton, Maryland, USA, 2016. **1**.

19.	Tegegne, T.K., et al., *Spatial and hierarchical Bayesian analysis to identify factors associated with caesarean delivery use in Ethiopia: Evidence from national population and health facility data.* Plos one, 2022. **17**(11): p. e0277885.

20.	Gebremedhin, S., *Trend and socio-demographic differentials of Caesarean section rate in Addis Ababa, Ethiopia: analysis based on Ethiopia demographic and health surveys data.* Reproductive health, 2014. **11**(1): p. 1-6.

21.	Welay, F.T., et al., *Delivery mode preference and associated factors among pregnant mothers in Harar regional state, Eastern Ethiopia: a cross-sectional study.* BioMed Research International, 2021. **2021**.

22.	Ryding, E.L., et al., *Pregnant women’s preference for cesarean section and subsequent mode of birth–a six-country cohort study.* Journal of Psychosomatic Obstetrics & Gynecology, 2016. **37**(3): p. 75-83.

23.	Løvåsmoen, E.M.L., et al., *Women’s preference for caesarean section and the actual mode of delivery–comparing five sites in Norway.* Sexual & reproductive healthcare, 2018. **16**: p. 206-212.

24.	Tenaw, Z., et al., *Maternal preference, mode of delivery and associated factors among women who gave birth at public and private hospitals in Hawassa City, Southern Ethiopia.* Annals of Global Health, 2019. **85**(1).

25.	Eide, K.T. and K. Bærøe, *How to reach trustworthy decisions for caesarean sections on maternal request: a call for beneficial power.* Journal of Medical Ethics, 2021. **47**(12): p. e45-e45.

26.	Ghotbi, F., et al., *Women's knowledge and attitude towards mode of delivery and frequency of cesarean section on mother's request in six public and private hospitals in T ehran, I ran, 2012.* Journal of Obstetrics and Gynaecology Research, 2014. **40**(5): p. 1257-1266.

27.	Misaeli, C., et al., *Factors associated with women’s intention to request caesarean delivery in Dar es Salaam, Tanzania.* South African Journal of Obstetrics and Gynaecology, 2017. **23**(2): p. 56-62.

28.	Joshi, A., M. Thapa, and O.B. Panta, *Maternal attitude and knowledge towards modes of delivery.* Journal of Nepal Health Research Council, 2018. **16**(2): p. 209-214.

29.	Karoni, H.F., et al., *Maternal satisfaction among vaginal and cesarean section delivery care services in Bahir Dar city health facilities, Northwest Ethiopia: a facility-based comparative cross-sectional study.* BMC pregnancy and childbirth, 2020. **20**(1): p. 1-14.

30.	Yusuf, A., et al., *PREVALENCE OF CESARIAN DELIVERY AND ASSOCIATED FACTORS AMONG MOTHERS WHO GAVE BIRTH AT PUBLIC HOSPITALS, JIGJIGA, ETHIOPIA.* 2023.

31.	Zewude, B., G. Siraw, and Y. Adem, *The Preferences of Modes of Child Delivery and Associated Factors Among Pregnant Women in Southern Ethiopia.* Pragmatic and Observational Research, 2022: p. 59-73.

32.	Mohamed, S.L., H. Fouly, and M. Mohamed, *Comparative study between Assiut and Zagazig regarding women preferences for mode of delivery.* IOSR Journal of Nursing and Health Science (IOSR-JNHS), 2016. **5**(3): p. 35-45.

33.	Kidane, A., et al., *Maternal satisfaction on delivery care services and associated factors at public hospitals in eastern Ethiopia.* International Health, 2023. **15**(2): p. 189-197.

34.	Venugopala, K., V. Rashmi, and B. Odhav, *BioMed Res. Intl.* Article ID, 2013. **963248**: p. 14.

35.	Tesfaye, R., et al., *Client satisfaction with delivery care service and associated factors in the public health facilities of Gamo Gofa zone, Southwest Ethiopia: in a resource limited setting.* Obstetrics and gynecology international, 2016. **2016**.

36.	Maharlouei, N., et al., *The preference of Iranian women to have normal vaginal or cesarean deliveries.* Journal of research in medical sciences: the official journal of Isfahan University of Medical Sciences, 2013. **18**(11): p. 943.

37.	Neuman, M., et al., *Prevalence and determinants of caesarean section in private and public health facilities in underserved South Asian communities: cross-sectional analysis of data from Bangladesh, India and Nepal.* BMJ open, 2014. **4**(12): p. e005982.

38.	Parvej, M.I., M. Tabassum, and N. Aktar, *Preferences between Caesarean Section and Normal Vaginal Delivery among the reproductive women in Bangladesh.* Journal of Applied Science, Engineering, Technology, and Education, 2021. **3**(1): p. 82-89.

39.	Adu-Bonsaffoh, K., E. Tamma, and J. Seffah, *Preferred mode of childbirth among women attending antenatal clinic at a tertiary hospital in Ghana: a cross-sectional study.* African Health Sciences, 2022. **22**(2): p. 480-488.

40.	Aziken, M., L. Omo-Aghoja, and F. Okonofua, *Perceptions and attitudes of pregnant women towards caesarean section in urban Nigeria.* Acta obstetricia et gynecologica Scandinavica, 2007. **86**(1): p. 42-47.
