# Supplementary material for: Preference of cesarean delivery and its associated factors among pregnant women attending ante natal care at public health facilities of Debrebrehan City, Ethiopia: Cross-sectional study
Source: PLoS One. 2024 Jan 31;19(1):e0296990. doi: 10.1371/journal.pone.0296990 (PMC10829985; doi:10.1371/journal.pone.0296990)
Supplement: Attachment — Submitted filename: Response to Reviewers.docx [file pone.0296990.s008.docx]

**RESPONSE TO EDITOR AND REVIEWERS**

**RESPONSE TO EDITOR**

**RESPONSE:** Thank you for coordinating the review process and fruitful comments. We have revised the manuscript and addressed Reviewer’s comments.

1. If the authors have adequately addressed your comments raised in a previous round of review and you feel that this manuscript is now acceptable for publication, you may indicate that here to bypass the “Comments to the Author” section, enter your conflict of interest statement in the “Confidential to Editor” section, and submit your "Accept" recommendation.

Reviewer #3: All comments have been addressed

Reviewer #4: (No Response)

##### RESPONSE: we try to address all comments.

2. Is the manuscript technically sound, and do the data support the conclusions?

The manuscript must describe a technically sound piece of scientific research with data that supports the conclusions. Experiments must have been conducted rigorously, with appropriate controls, replication, and sample sizes. The conclusions must be drawn appropriately based on the data presented.

Reviewer #3: Yes

Reviewer #4: Partly

**RESPONSE:** Now we made and corrected all comments.

3. Has the statistical analysis been performed appropriately and rigorously?

Reviewer #3: Yes

Reviewer #4: Yes

**RESPONSE:** N/A

4. Have the authors made all data underlying the findings in their manuscript fully available?

The [PLOS Data policy](http://www.plosone.org/static/policies.action#sharing) requires authors to make all data underlying the findings described in their manuscript fully available without restriction, with rare exception (please refer to the Data Availability Statement in the manuscript PDF file). The data should be provided as part of the manuscript or its supporting information, or deposited to a public repository. For example, in addition to summary statistics, the data points behind means, medians and variance measures should be available. If there are restrictions on publicly sharing data—e.g. participant privacy or use of data from a third party—those must be specified.

Reviewer #3: Yes

Reviewer #4: Yes

**RESPONSE:** N/A

5. Is the manuscript presented in an intelligible fashion and written in standard English?

PLOS ONE does not copyedit accepted manuscripts, so the language in submitted articles must be clear, correct, and unambiguous. Any typographical or grammatical errors should be corrected at revision, so please note any specific errors here.

Reviewer #3: Yes

Reviewer #4: No

**RESPONSE:** We revise and try to rewrite some language error.

6. Review Comments to the Author

Please use the space provided to explain your answers to the questions above. You may also include additional comments for the author, including concerns about dual publication, research ethics, or publication ethics. (Please upload your review as an attachment if it exceeds 20,000 characters)

Reviewer #3: The author addressed the comments as expected and improved the manuscript significantly. However, I would request the author address the few comments and the paper can be accepted, mostly grammatical and formatting issues

Reviewer #4: The authors conducted cross sectional study to determine preference of caesarean delivery among pregnant women in Debre Berhan city. They have recruited 512 participants with multistage sampling. My comments here
1. single population proportion formula was used to calculate the sample size; however, the study was exploring factors associated with preference for caesarean delivery. Here the sample size for the factors was not estimated yet multivariate logistic regression was fitted. Hence adequately representative sample may not be used.

**RESPONSE:** **Thank you for your interesting comments. We made second objective sample size calculation but it was low sample size than single population proportion formula (here is attached see the table).**

| Variable | Proportion out come | AOR | Sample size | After adding 10% | After multiplying 1.5 |
| --- | --- | --- | --- | --- | --- |
| Age | P1=0.2412  P2=0.0025 | 2.9 | 74 | 82 | 123 |
| Gravidity | P1=0.128  P2=0.015 | 1.24 | 196 | 216 | 324 |
| Birth place preference | P1=0.264  P2=0.023 | 2.2 | 80 | 88 | 132 |
| Planned pregnancy | P1=0.206  P2=0.083 | 1.76 | 288 | 316.8 | 475 |

2. The exclusion criteria:
how was the outcome of previous pregnancy considered?
what was the Gestational age limit used to delineate between 1st, 2nd and 3rd trimester.
How was the Gestational age assessment?
how were women with prior home delivery entertained?

RESPONSE: In this study, we excluded only women who had previous c/s scares; we did not consider the outcome, site of delivery (home delivery), or GA because our objective is to determine their preference for delivery.

3. In the knowledge assessment there is inconsistent use of terms like poor knowledge, no knowledge,

**RESPONSE: we accepted and amend it accordingly.**

4. The information, counselling given Durning ANC varies by trimester and gestational age i.e. mode of delivery often discussed in the third trimester, that could potentially affect the knowledge on caesarean section. How do you see treating (including in the study) participants in the first and third trimester, those with first ANC contact with those having more than one ANC Contact.

**RESPONSE:** **We appreciate your view. But, in our consideration, a woman, after first and second contact, may change their previous preference duo to counseling’s during ANC follow-up. Due to this, their number of ANC visits did not have an association with dependent variables.**

5. The authors didn't mention the limitation of the study, could you elaborate.

**RESPONSE: we accepted and amend it accordingly.**
6. There are flaws in the Language used, I recommend looking into it again

**RESPONSE: we accepted and amend it accordingly.**

7. PLOS authors have the option to publish the peer review history of their article ([what does this mean?](https://journals.plos.org/plosone/s/editorial-and-peer-review-process#loc-peer-review-history)). If published, this will include your full peer review and any attached files. If you choose “no”, your identity will remain anonymous but your review may still be made public.

**Do you want your identity to be public for this peer review?** For information about this choice, including consent withdrawal, please see our [Privacy Policy](https://www.plos.org/privacy-policy).

Reviewer #3: **Yes:** Emmanuel Ekung

Reviewer #4: No

We thanks too!
